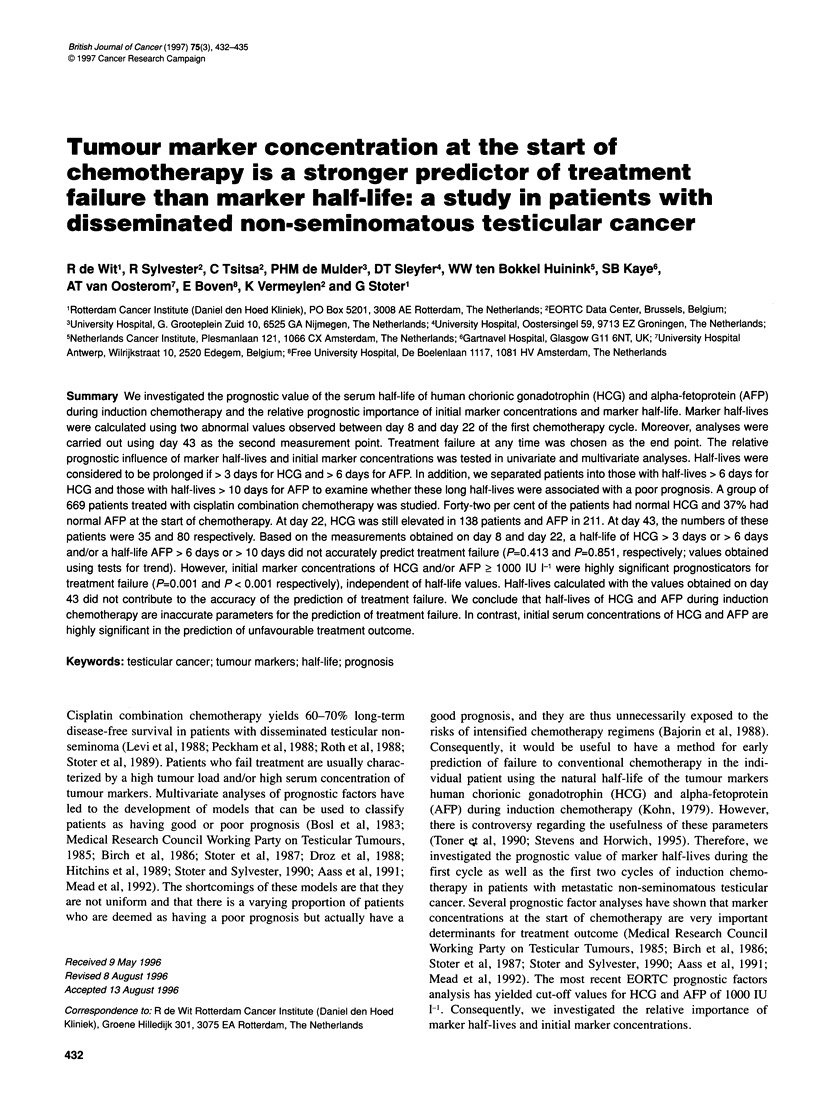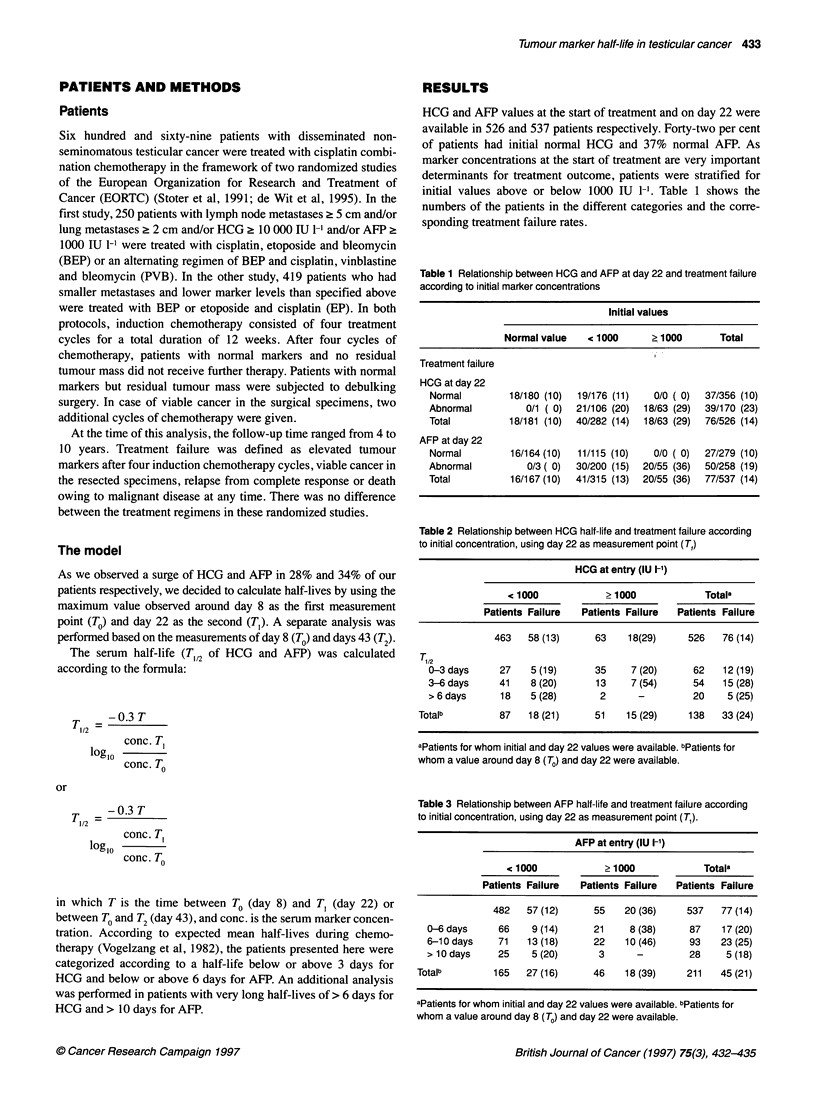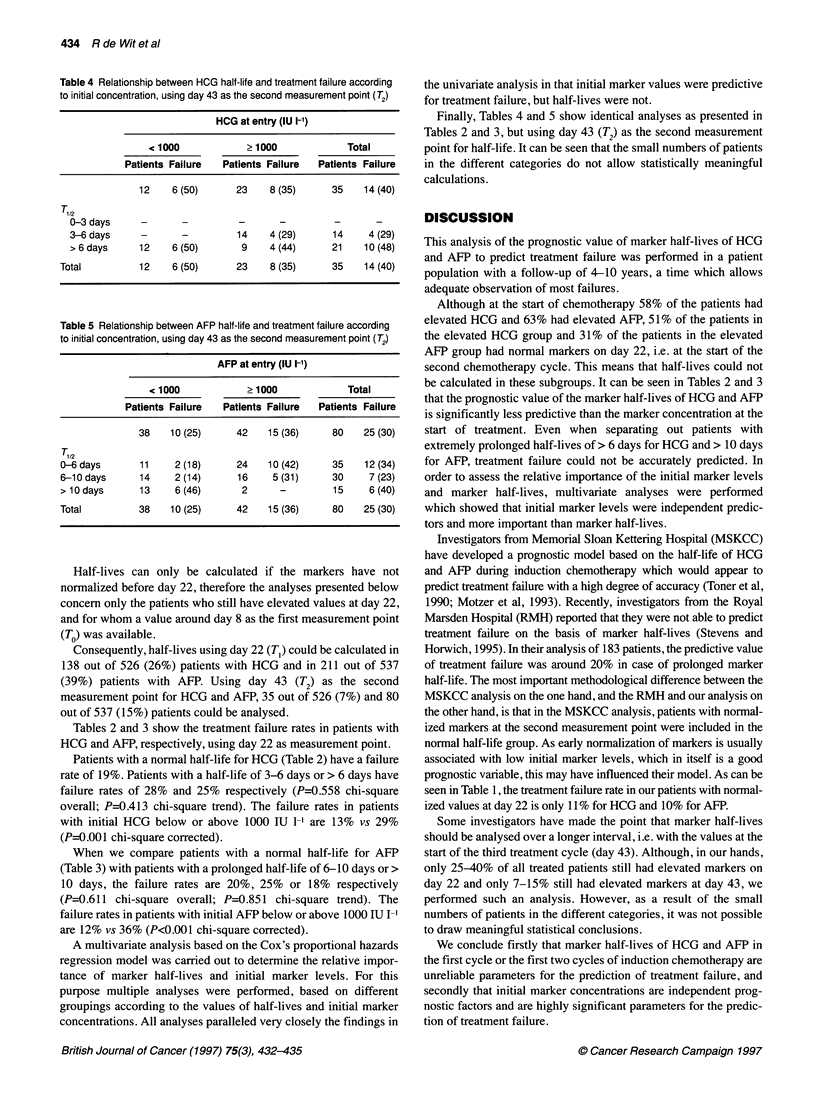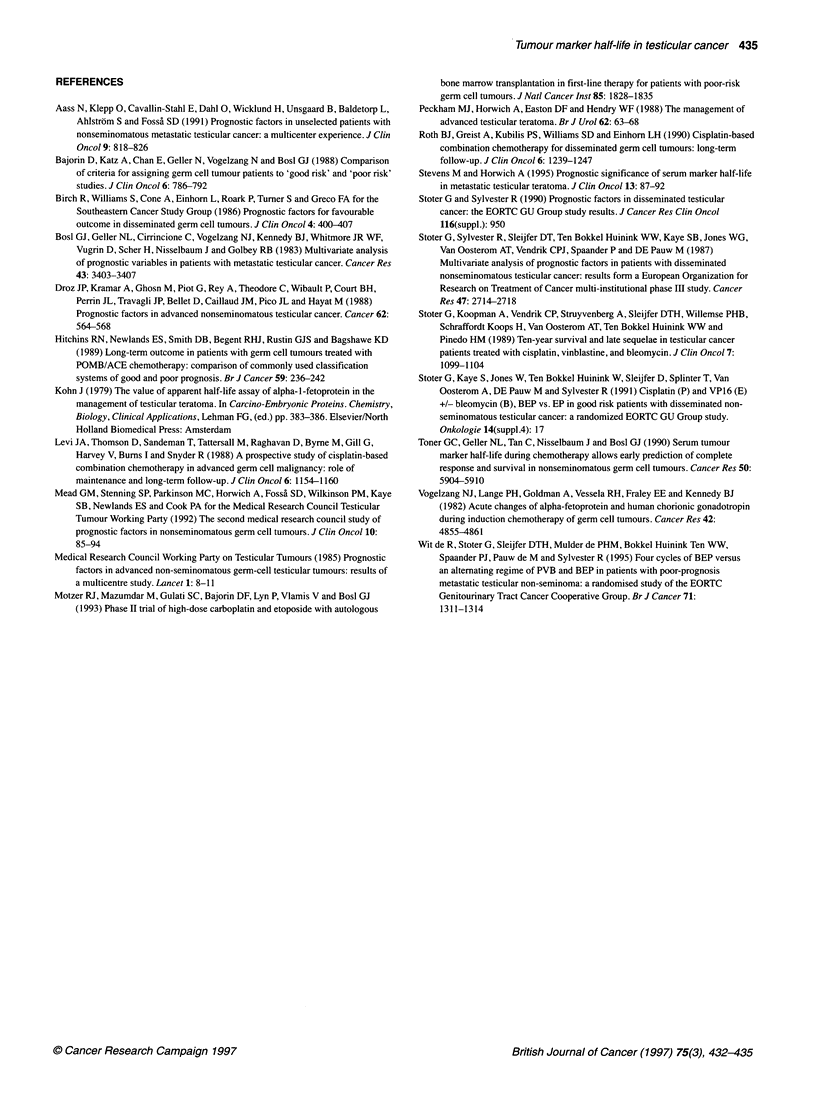# Tumour marker concentration at the start of chemotherapy is a stronger predictor of treatment failure than marker half-life: a study in patients with disseminated non-seminomatous testicular cancer.

**DOI:** 10.1038/bjc.1997.71

**Published:** 1997

**Authors:** R. de Wit, R. Sylvester, C. Tsitsa, P. H. de Mulder, D. T. Sleyfer, W. W. ten Bokkel Huinink, S. B. Kaye, A. T. van Oosterom, E. Boven, K. Vermeylen, G. Stoter

**Affiliations:** Rotterdam Cancer Institute (Daniel den Hoed Kliniek), The Netherlands.

## Abstract

We investigated the prognostic value of the serum half-life of human chorionic gonadotrophin (HCG) and alpha-fetoprotein (AFP) during induction chemotherapy and the relative prognostic importance of initial marker concentrations and marker half-life. Marker half-lives were calculated using two abnormal values observed between day 8 and day 22 of the first chemotherapy cycle. Moreover, analyses were carried out using day 43 as the second measurement point. Treatment failure at any time was chosen as the end point. The relative prognostic influence of marker half-lives and initial marker concentrations was tested in univariate and multivariate analyses. Half-lives were considered to be prolonged if > 3 days for HCG and > 6 days for AFP. In addition, we separated patients into those with half-lives > 6 days for HCG and those with half-lives > 10 days for AFP to examine whether these long half-lives were associated with a poor prognosis. A group of 669 patients treated with cisplatin combination chemotherapy was studied. Forty-two per cent of the patients had normal HCG and 37% had normal AFP at the start of chemotherapy. At day 22, HCG was still elevated in 138 patients and AFP in 211. At day 43, the numbers of these patients were 35 and 80 respectively. Based on the measurements obtained on day 8 and day 22, a half-life of HCG > 3 days or > 6 days and/or a half-life AFP > 6 days or > 10 days did not accurately predict treatment failure (P=0.413 and P=0.851, respectively; values obtained using tests for trend). However, initial marker concentrations of HCG and/or AFP > 1000 IU l(-1) were highly significant prognosticators for treatment failure (P=0.001 and P < 0.001 respectively), independent of half-life values. Half-lives calculated with the values obtained on day 43 did not contribute to the accuracy of the prediction of treatment failure. We conclude that half-lives of HCG and AFP during induction chemotherapy are inaccurate parameters for the prediction of treatment failure. In contrast, initial serum concentrations of HCG and AFP are highly significant in the prediction of unfavourable treatment outcome.


British Journal of Cancer (1997) 75(3), 432–435
© 1997 Cancer Research Campaign

### Tumour marker concentration at the start of chemotherapy is a stronger predictor of treatment failure than marker half-life: a study in patients with disseminated non-seminomatous testicular cancer

**R de Wit[1], R Sylvester[2], C Tsitsa[2], PHM de Mulder[3], DT Sleyfer[4], WW ten Bokkel Huinink[5], SB Kaye[6], AT van Oosterom[7], E Boven[8], K Vermeylen[2] and G Stoter[1]**

[1]Rotterdam Cancer Institute (Daniel den Hoed Kliniek), PO Box 5201, 3008 AE Rotterdam, The Netherlands; [2]EORTC Data Center, Brussels, Belgium; [3]University Hospital, G. Grooteplein Zuid 10, 6525 GA Nijmegen, The Netherlands; [4]University Hospital, Oostersingel 59, 9713 EZ Groningen, The Netherlands; [5]Netherlands Cancer Institute, Plesmanlaan 121, 1066 CX Amsterdam, The Netherlands; [6]Gartnavel Hospital, Glasgow G11 6NT, UK; [7]University Hospital Antwerp, Wilrijkstraat 10, 2520 Edegem, Belgium; [8]Free University Hospital, De Boelenlaan 1117, 1081 HV Amsterdam, The Netherlands

**Summary** We investigated the prognostic value of the serum half-life of human chorionic gonadotrophin (HCG) and alpha-fetoprotein (AFP) during induction chemotherapy and the relative prognostic importance of initial marker concentrations and marker half-life. Marker half-lives were calculated using two abnormal values observed between day 8 and day 22 of the first chemotherapy cycle. Moreover, analyses were carried out using day 43 as the second measurement point. Treatment failure at any time was chosen as the end point. The relative prognostic influence of marker half-lives and initial marker concentrations was tested in univariate and multivariate analyses. Half-lives were considered to be prolonged if > 3 days for HCG and > 6 days for AFP. In addition, we separated patients into those with half-lives > 6 days for HCG and those with half-lives > 10 days for AFP to examine whether these long half-lives were associated with a poor prognosis. A group of 669 patients treated with cisplatin combination chemotherapy was studied. Forty-two per cent of the patients had normal HCG and 37% had normal AFP at the start of chemotherapy. At day 22, HCG was still elevated in 138 patients and AFP in 211. At day 43, the numbers of these patients were 35 and 80 respectively. Based on the measurements obtained on day 8 and day 22, a half-life of HCG > 3 days or > 6 days and/or a half-life AFP > 6 days or > 10 days did not accurately predict treatment failure ($P$=0.413 and $P$=0.851, respectively; values obtained using tests for trend). However, initial marker concentrations of HCG and/or AFP ≥ 1000 IU $l^{-1}$ were highly significant prognosticators for treatment failure ($P$=0.001 and $P < 0.001$ respectively), independent of half-life values. Half-lives calculated with the values obtained on day 43 did not contribute to the accuracy of the prediction of treatment failure. We conclude that half-lives of HCG and AFP during induction chemotherapy are inaccurate parameters for the prediction of treatment failure. In contrast, initial serum concentrations of HCG and AFP are highly significant in the prediction of unfavourable treatment outcome.

**Keywords:** testicular cancer; tumour markers; half-life; prognosis

Cisplatin combination chemotherapy yields 60–70% long-term disease-free survival in patients with disseminated testicular non-seminoma (Levi et al, 1988; Peckham et al, 1988; Roth et al, 1988; Stoter et al, 1989). Patients who fail treatment are usually characterized by a high tumour load and/or high serum concentration of tumour markers. Multivariate analyses of prognostic factors have led to the development of models that can be used to classify patients as having good or poor prognosis (Bosl et al, 1983; Medical Research Council Working Party on Testicular Tumours, 1985; Birch et al, 1986; Stoter et al, 1987; Droz et al, 1988; Hitchins et al, 1989; Stoter and Sylvester, 1990; Aass et al, 1991; Mead et al, 1992). The shortcomings of these models are that they are not uniform and that there is a varying proportion of patients who are deemed as having a poor prognosis but actually have a good prognosis, and they are thus unnecessarily exposed to the risks of intensified chemotherapy regimens (Bajorin et al, 1988). Consequently, it would be useful to have a method for early prediction of failure to conventional chemotherapy in the individual patient using the natural half-life of the tumour markers human chorionic gonadotrophin (HCG) and alpha-fetoprotein (AFP) during induction chemotherapy (Kohn, 1979). However, there is controversy regarding the usefulness of these parameters (Toner et al, 1990; Stevens and Horwich, 1995). Therefore, we investigated the prognostic value of marker half-lives during the first cycle as well as the first two cycles of induction chemotherapy in patients with metastatic non-seminomatous testicular cancer. Several prognostic factor analyses have shown that marker concentrations at the start of chemotherapy are very important determinants for treatment outcome (Medical Research Council Working Party on Testicular Tumours, 1985; Birch et al, 1986; Stoter et al, 1987; Stoter and Sylvester, 1990; Aass et al, 1991; Mead et al, 1992). The most recent EORTC prognostic factors analysis has yielded cut-off values for HCG and AFP of 1000 IU $l^{-1}$. Consequently, we investigated the relative importance of marker half-lives and initial marker concentrations.

*Received 9 May 1996*
*Revised 8 August 1996*
*Accepted 13 August 1996*

*Correspondence to:* R de Wit Rotterdam Cancer Institute (Daniel den Hoed Kliniek), Groene Hilledijk 301, 3075 EA Rotterdam, The Netherlands

### PATIENTS AND METHODS

### Patients

Six hundred and sixty-nine patients with disseminated non-seminomatous testicular cancer were treated with cisplatin combination chemotherapy in the framework of two randomized studies of the European Organization for Research and Treatment of Cancer (EORTC) (Stoter et al, 1991; de Wit et al, 1995). In the first study, 250 patients with lymph node metastases ≥ 5 cm and/or lung metastases ≥ 2 cm and/or HCG ≥ 10 000 IU $l^{-1}$ and/or AFP ≥ 1000 IU $l^{-1}$ were treated with cisplatin, etoposide and bleomycin (BEP) or an alternating regimen of BEP and cisplatin, vinblastine and bleomycin (PVB). In the other study, 419 patients who had smaller metastases and lower marker levels than specified above were treated with BEP or etoposide and cisplatin (EP). In both protocols, induction chemotherapy consisted of four treatment cycles for a total duration of 12 weeks. After four cycles of chemotherapy, patients with normal markers and no residual tumour mass did not receive further therapy. Patients with normal markers but residual tumour mass were subjected to debulking surgery. In case of viable cancer in the surgical specimens, two additional cycles of chemotherapy were given.

At the time of this analysis, the follow-up time ranged from 4 to 10 years. Treatment failure was defined as elevated tumour markers after four induction chemotherapy cycles, viable cancer in the resected specimens, relapse from complete response or death owing to malignant disease at any time. There was no difference between the treatment regimens in these randomized studies.

### The model

As we observed a surge of HCG and AFP in 28% and 34% of our patients respectively, we decided to calculate half-lives by using the maximum value observed around day 8 as the first measurement point ($T_0$) and day 22 as the second ($T_1$). A separate analysis was performed based on the measurements of day 8 ($T_0$) and days 43 ($T_2$).

The serum half-life ($T_{1/2}$ of HCG and AFP) was calculated according to the formula:

$$T_{1/2} = \frac{-0.3\,T}{\log_{10} \dfrac{\text{conc. } T_1}{\text{conc. } T_0}}$$

or

$$T_{1/2} = \frac{-0.3\,T}{\log_{10} \dfrac{\text{conc. } T_1}{\text{conc. } T_0}}$$

in which $T$ is the time between $T_0$ (day 8) and $T_1$ (day 22) or between $T_0$ and $T_2$ (day 43), and conc. is the serum marker concentration. According to expected mean half-lives during chemotherapy (Vogelzang et al, 1982), the patients presented here were categorized according to a half-life below or above 3 days for HCG and below or above 6 days for AFP. An additional analysis was performed in patients with very long half-lives of > 6 days for HCG and > 10 days for AFP.

### RESULTS

HCG and AFP values at the start of treatment and on day 22 were available in 526 and 537 patients respectively. Forty-two per cent of patients had initial normal HCG and 37% normal AFP. As marker concentrations at the start of treatment are very important determinants for treatment outcome, patients were stratified for initial values above or below 1000 IU $l^{-1}$. Table 1 shows the numbers of the patients in the different categories and the corresponding treatment failure rates.

**Table 1** Relationship between HCG and AFP at day 22 and treatment failure according to initial marker concentrations

| | Initial values | | | |
|---|---|---|---|---|
| | Normal value | < 1000 | ≥ 1000 | Total |
| Treatment failure | | | | |
| HCG at day 22 | | | | |
| Normal | 18/180 (10) | 19/176 (11) | 0/0 ( 0) | 37/356 (10) |
| Abnormal | 0/1 ( 0) | 21/106 (20) | 18/63 (29) | 39/170 (23) |
| Total | 18/181 (10) | 40/282 (14) | 18/63 (29) | 76/526 (14) |
| AFP at day 22 | | | | |
| Normal | 16/164 (10) | 11/115 (10) | 0/0 ( 0) | 27/279 (10) |
| Abnormal | 0/3 ( 0) | 30/200 (15) | 20/55 (36) | 50/258 (19) |
| Total | 16/167 (10) | 41/315 (13) | 20/55 (36) | 77/537 (14) |

**Table 2** Relationship between HCG half-life and treatment failure according to initial concentration, using day 22 as measurement point ($T_1$)

| | HCG at entry (IU $l^{-1}$) | | | | | |
|---|---|---|---|---|---|---|
| | < 1000 | | ≥ 1000 | | Total[a] | |
| | Patients | Failure | Patients | Failure | Patients | Failure |
| | 463 | 58 (13) | 63 | 18(29) | 526 | 76 (14) |
| $T_{1/2}$ | | | | | | |
| 0–3 days | 27 | 5 (19) | 35 | 7 (20) | 62 | 12 (19) |
| 3–6 days | 41 | 8 (20) | 13 | 7 (54) | 54 | 15 (28) |
| > 6 days | 18 | 5 (28) | 2 | – | 20 | 5 (25) |
| Total[b] | 87 | 18 (21) | 51 | 15 (29) | 138 | 33 (24) |

[a]Patients for whom initial and day 22 values were available. [b]Patients for whom a value around day 8 ($T_0$) and day 22 were available.

**Table 3** Relationship between AFP half-life and treatment failure according to initial concentration, using day 22 as measurement point ($T_1$).

| | AFP at entry (IU $l^{-1}$) | | | | | |
|---|---|---|---|---|---|---|
| | < 1000 | | ≥ 1000 | | Total[a] | |
| | Patients | Failure | Patients | Failure | Patients | Failure |
| | 482 | 57 (12) | 55 | 20 (36) | 537 | 77 (14) |
| 0–6 days | 66 | 9 (14) | 21 | 8 (38) | 87 | 17 (20) |
| 6–10 days | 71 | 13 (18) | 22 | 10 (46) | 93 | 23 (25) |
| > 10 days | 25 | 5 (20) | 3 | – | 28 | 5 (18) |
| Total[b] | 165 | 27 (16) | 46 | 18 (39) | 211 | 45 (21) |

[a]Patients for whom initial and day 22 values were available. [b]Patients for whom a value around day 8 ($T_0$) and day 22 were available.

© Cancer Research Campaign 1997

**Table 4** Relationship between HCG half-life and treatment failure according to initial concentration, using day 43 as the second measurement point ($T_2$)

| | HCG at entry (IU l⁻¹) | | | | | |
|---|---|---|---|---|---|---|
| | < 1000 | | ≥ 1000 | | Total | |
| | Patients | Failure | Patients | Failure | Patients | Failure |
| | 12 | 6 (50) | 23 | 8 (35) | 35 | 14 (40) |
| $T_{1/2}$ | | | | | | |
| 0–3 days | – | – | – | – | – | – |
| 3–6 days | – | – | 14 | 4 (29) | 14 | 4 (29) |
| > 6 days | 12 | 6 (50) | 9 | 4 (44) | 21 | 10 (48) |
| Total | 12 | 6 (50) | 23 | 8 (35) | 35 | 14 (40) |

**Table 5** Relationship between AFP half-life and treatment failure according to initial concentration, using day 43 as the second measurement point ($T_2$)

| | AFP at entry (IU l⁻¹) | | | | | |
|---|---|---|---|---|---|---|
| | < 1000 | | ≥ 1000 | | Total | |
| | Patients | Failure | Patients | Failure | Patients | Failure |
| | 38 | 10 (25) | 42 | 15 (36) | 80 | 25 (30) |
| $T_{1/2}$ | | | | | | |
| 0–6 days | 11 | 2 (18) | 24 | 10 (42) | 35 | 12 (34) |
| 6–10 days | 14 | 2 (14) | 16 | 5 (31) | 30 | 7 (23) |
| > 10 days | 13 | 6 (46) | 2 | – | 15 | 6 (40) |
| Total | 38 | 10 (25) | 42 | 15 (36) | 80 | 25 (30) |

Half-lives can only be calculated if the markers have not normalized before day 22, therefore the analyses presented below concern only the patients who still have elevated values at day 22, and for whom a value around day 8 as the first measurement point ($T_0$) was available.

Consequently, half-lives using day 22 ($T_1$) could be calculated in 138 out of 526 (26%) patients with HCG and in 211 out of 537 (39%) patients with AFP. Using day 43 ($T_2$) as the second measurement point for HCG and AFP, 35 out of 526 (7%) and 80 out of 537 (15%) patients could be analysed.

Tables 2 and 3 show the treatment failure rates in patients with HCG and AFP, respectively, using day 22 as measurement point.

Patients with a normal half-life for HCG (Table 2) have a failure rate of 19%. Patients with a half-life of 3–6 days or > 6 days have failure rates of 28% and 25% respectively ($P$=0.558 chi-square overall; $P$=0.413 chi-square trend). The failure rates in patients with initial HCG below or above 1000 IU $l^{-1}$ are 13% *vs* 29% ($P$=0.001 chi-square corrected).

When we compare patients with a normal half-life for AFP (Table 3) with patients with a prolonged half-life of 6–10 days or > 10 days, the failure rates are 20%, 25% or 18% respectively ($P$=0.611 chi-square overall; $P$=0.851 chi-square trend). The failure rates in patients with initial AFP below or above 1000 IU $l^{-1}$ are 12% *vs* 36% ($P$<0.001 chi-square corrected).

A multivariate analysis based on the Cox's proportional hazards regression model was carried out to determine the relative importance of marker half-lives and initial marker levels. For this purpose multiple analyses were performed, based on different groupings according to the values of half-lives and initial marker concentrations. All analyses paralleled very closely the findings in the univariate analysis in that initial marker values were predictive for treatment failure, but half-lives were not.

Finally, Tables 4 and 5 show identical analyses as presented in Tables 2 and 3, but using day 43 ($T_2$) as the second measurement point for half-life. It can be seen that the small numbers of patients in the different categories do not allow statistically meaningful calculations.

### DISCUSSION

This analysis of the prognostic value of marker half-lives of HCG and AFP to predict treatment failure was performed in a patient population with a follow-up of 4–10 years, a time which allows adequate observation of most failures.

Although at the start of chemotherapy 58% of the patients had elevated HCG and 63% had elevated AFP, 51% of the patients in the elevated HCG group and 31% of the patients in the elevated AFP group had normal markers on day 22, i.e. at the start of the second chemotherapy cycle. This means that half-lives could not be calculated in these subgroups. It can be seen in Tables 2 and 3 that the prognostic value of the marker half-lives of HCG and AFP is significantly less predictive than the marker concentration at the start of treatment. Even when separating out patients with extremely prolonged half-lives of > 6 days for HCG and > 10 days for AFP, treatment failure could not be accurately predicted. In order to assess the relative importance of the initial marker levels and marker half-lives, multivariate analyses were performed which showed that initial marker levels were independent predictors and more important than marker half-lives.

Investigators from Memorial Sloan Kettering Hospital (MSKCC) have developed a prognostic model based on the half-life of HCG and AFP during induction chemotherapy which would appear to predict treatment failure with a high degree of accuracy (Toner et al, 1990; Motzer et al, 1993). Recently, investigators from the Royal Marsden Hospital (RMH) reported that they were not able to predict treatment failure on the basis of marker half-lives (Stevens and Horwich, 1995). In their analysis of 183 patients, the predictive value of treatment failure was around 20% in case of prolonged marker half-life. The most important methodological difference between the MSKCC analysis on the one hand, and the RMH and our analysis on the other hand, is that in the MSKCC analysis, patients with normalized markers at the second measurement point were included in the normal half-life group. As early normalization of markers is usually associated with low initial marker levels, which in itself is a good prognostic variable, this may have influenced their model. As can be seen in Table 1, the treatment failure rate in our patients with normalized values at day 22 is only 11% for HCG and 10% for AFP.

Some investigators have made the point that marker half-lives should be analysed over a longer interval, i.e. with the values at the start of the third treatment cycle (day 43). Although, in our hands, only 25–40% of all treated patients still had elevated markers on day 22 and only 7–15% still had elevated markers at day 43, we performed such an analysis. However, as a result of the small numbers of patients in the different categories, it was not possible to draw meaningful statistical conclusions.

We conclude firstly that marker half-lives of HCG and AFP in the first cycle or the first two cycles of induction chemotherapy are unreliable parameters for the prediction of treatment failure, and secondly that initial marker concentrations are independent prognostic factors and are highly significant parameters for the prediction of treatment failure.

© Cancer Research Campaign 1997

### REFERENCES

Aass N, Klepp O, Cavallin-Stahl E, Dahl O, Wicklund H, Unsgaard B, Baldetorp L, Ahlström S and Fosså SD (1991) Prognostic factors in unselected patients with nonseminomatous metastatic testicular cancer: a multicenter experience. *J Clin Oncol* **9**: 818–826

Bajorin D, Katz A, Chan E, Geller N, Vogelzang N and Bosl GJ (1988) Comparison of criteria for assigning germ cell tumour patients to 'good risk' and 'poor risk' studies. *J Clin Oncol* **6**: 786–792

Birch R, Williams S, Cone A, Einhorn L, Roark P, Turner S and Greco FA for the Southeastern Cancer Study Group (1986) Prognostic factors for favourable outcome in disseminated germ cell tumours. *J Clin Oncol* **4**: 400–407

Bosl GJ, Geller NL, Cirrincione C, Vogelzang NJ, Kennedy BJ, Whitmore JR WF, Vugrin D, Scher H, Nisselbaum J and Golbey RB (1983) Multivariate analysis of prognostic variables in patients with metastatic testicular cancer. *Cancer Res* **43**: 3403–3407

Droz JP, Kramar A, Ghosn M, Piot G, Rey A, Theodore C, Wibault P, Court BH, Perrin JL, Travagli JP, Bellet D, Caillaud JM, Pico JL and Hayat M (1988) Prognostic factors in advanced nonseminomatous testicular cancer. *Cancer* **62**: 564–568

Hitchins RN, Newlands ES, Smith DB, Begent RHJ, Rustin GJS and Bagshawe KD (1989) Long-term outcome in patients with germ cell tumours treated with POMB/ACE chemotherapy: comparison of commonly used classification systems of good and poor prognosis. *Br J Cancer* **59**: 236–242

Kohn J (1979) The value of apparent half-life assay of alpha-1-fetoprotein in the management of testicular teratoma. In *Carcino-Embryonic Proteins. Chemistry, Biology, Clinical Applications*, Lehman FG, (ed.) pp. 383–386. Elsevier/North Holland Biomedical Press: Amsterdam

Levi JA, Thomson D, Sandeman T, Tattersall M, Raghavan D, Byrne M, Gill G, Harvey V, Burns I and Snyder R (1988) A prospective study of cisplatin-based combination chemotherapy in advanced germ cell malignancy: role of maintenance and long-term follow-up. *J Clin Oncol* **6**: 1154–1160

Mead GM, Stenning SP, Parkinson MC, Horwich A, Fosså SD, Wilkinson PM, Kaye SB, Newlands ES and Cook PA for the Medical Research Council Testicular Tumour Working Party (1992) The second medical research council study of prognostic factors in nonseminomatous germ cell tumours. *J Clin Oncol* **10**: 85–94

Medical Research Council Working Party on Testicular Tumours (1985) Prognostic factors in advanced non-seminomatous germ-cell testicular tumours: results of a multicentre study. *Lancet* **1**: 8–11

Motzer RJ, Mazumdar M, Gulati SC, Bajorin DF, Lyn P, Vlamis V and Bosl GJ (1993) Phase II trial of high-dose carboplatin and etoposide with autologous bone marrow transplantation in first-line therapy for patients with poor-risk germ cell tumours. *J Natl Cancer Inst* **85**: 1828–1835

Peckham MJ, Horwich A, Easton DF and Hendry WF (1988) The management of advanced testicular teratoma. *Br J Urol* **62**: 63–68

Roth BJ, Greist A, Kubilis PS, Williams SD and Einhorn LH (1990) Cisplatin-based combination chemotherapy for disseminated germ cell tumours: long-term follow-up. *J Clin Oncol* **6**: 1239–1247

Stevens M and Horwich A (1995) Prognostic significance of serum marker half-life in metastatic testicular teratoma. *J Clin Oncol* **13**: 87–92

Stoter G and Sylvester R (1990) Prognostic factors in disseminated testicular cancer: the EORTC GU Group study results. *J Cancer Res Clin Oncol* **116**(suppl.): 950

Stoter G, Sylvester R, Sleijfer DT, Ten Bokkel Huinink WW, Kaye SB, Jones WG, Van Oosterom AT, Vendrik CPJ, Spaander P and DE Pauw M (1987) Multivariate analysis of prognostic factors in patients with disseminated nonseminomatous testicular cancer: results form a European Organization for Research on Treatment of Cancer multi-institutional phase III study. *Cancer Res* **47**: 2714–2718

Stoter G, Koopman A, Vendrik CP, Struyvenberg A, Sleijfer DTH, Willemse PHB, Schraffordt Koops H, Van Oosterom AT, Ten Bokkel Huinink WW and Pinedo HM (1989) Ten-year survival and late sequelae in testicular cancer patients treated with cisplatin, vinblastine, and bleomycin. *J Clin Oncol* **7**: 1099–1104

Stoter G, Kaye S, Jones W, Ten Bokkel Huinink W, Sleijfer D, Splinter T, Van Oosterom A, DE Pauw M and Sylvester R (1991) Cisplatin (P) and VP16 (E) +/− bleomycin (B), BEP vs. EP in good risk patients with disseminated non-seminomatous testicular cancer: a randomized EORTC GU Group study. *Onkologie* **14**(suppl.4): 17

Toner GC, Geller NL, Tan C, Nisselbaum J and Bosl GJ (1990) Serum tumour marker half-life during chemotherapy allows early prediction of complete response and survival in nonseminomatous germ cell tumours. *Cancer Res* **50**: 5904–5910

Vogelzang NJ, Lange PH, Goldman A, Vessela RH, Fraley EE and Kennedy BJ (1982) Acute changes of alpha-fetoprotein and human chorionic gonadotropin during induction chemotherapy of germ cell tumours. *Cancer Res* **42**: 4855–4861

Wit de R, Stoter G, Sleijfer DTH, Mulder de PHM, Bokkel Huinink Ten WW, Spaander PJ, Pauw de M and Sylvester R (1995) Four cycles of BEP versus an alternating regime of PVB and BEP in patients with poor-prognosis metastatic testicular non-seminoma: a randomised study of the EORTC Genitourinary Tract Cancer Cooperative Group. *Br J Cancer* **71**: 1311–1314

© Cancer Research Campaign 1997